# The Noxious Intruder of the Eye: Endogenous Klebsiella Panophthalmitis

**DOI:** 10.7759/cureus.18725

**Published:** 2021-10-12

**Authors:** Huei Xian Chai, Amir Samsudin, Kogilavaani Jayaraman, Mei Fong Chong

**Affiliations:** 1 Ophthalmology, University of Malaya Medical Centre, Selangor, MYS; 2 Ophthalmology, Hospital Raja Permaisuri Bainun, Ipoh, MYS

**Keywords:** evisceration, liver abscess, endogenous, panophthalmitis, klebsiella pneumoniae

## Abstract

Endogenous *Klebsiella pneumoniae* panophthalmitis commonly affects those with compromised immunity. The occurrence of this infection in healthy individuals is rare. We describe the case of a healthy adult who presented with endogenous *Klebsiella pneumoniae* panophthalmitis from an asymptomatic liver abscess.

A 64-year-old, previously healthy gentleman presented with rapidly progressive left eye periorbital swelling and blurring of vision. He had a low-grade fever three days prior to the development of ocular symptoms, but otherwise no other systemic complaints. Visual acuity was light perception in the left eye, and ocular motility was restricted in all directions of gaze. Ocular examination revealed proptosis, severe conjunctival chemosis, hazy cornea, and fibrin deposition in the anterior chamber. The posterior segment could not be visualized.

Ultrasound of the hepatobiliary system revealed an abscess in the right lobe of his liver. His blood cultures grew *Klebsiella pneumoniae*. Despite being treated with high-dose intravenous antibiotics, his eye condition deteriorated. Evisceration was performed when he developed scleral melting and globe perforation.

We highlight the importance of a high index of suspicion of endogenous *Klebsiella* panophthalmitis as it can be easily missed in healthy adults. Early diagnosis and prompt management are needed to prevent morbidity and mortality from this devastating infection.

## Introduction

Endogenous *Klebsiella pneumoniae* panophthalmitis is an emerging entity in South East Asia. It commonly affects those with compromised immunity, and the resultant visual outcome is usually poor. Prompt diagnosis with early identification of the source of infection followed by targeted treatment may, however, salvage useful vision. The occurrence of this infection in healthy individuals is rare and uncommon. We describe the case of a healthy adult who presented with endogenous *Klebsiella pneumoniae* panophthalmitis culminating in orbital apex syndrome and poor outcome for the eye.

This article was previously presented as a poster at the 35th Singapore-Malaysia Joint Meeting in Ophthalmology from January 17 to 19, 2020.

## Case presentation

**Figure 1:**
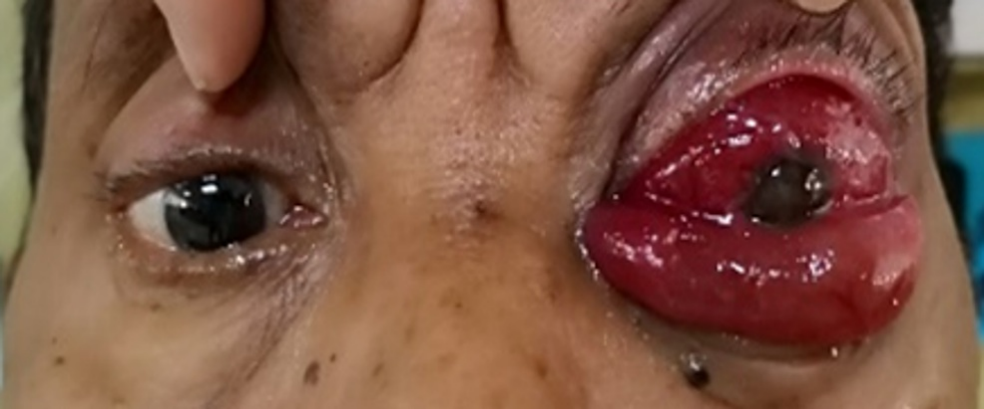
Patient presented with left eye proptosis with severe conjunctival chemosis on day 1 of admission.

**Figure 2:**
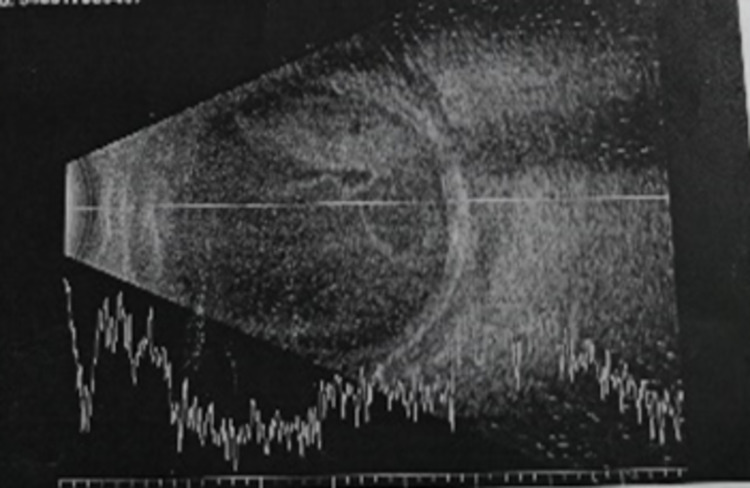
B-scan showed vitreous opacity and multiple loculations.

**Figure 3:**
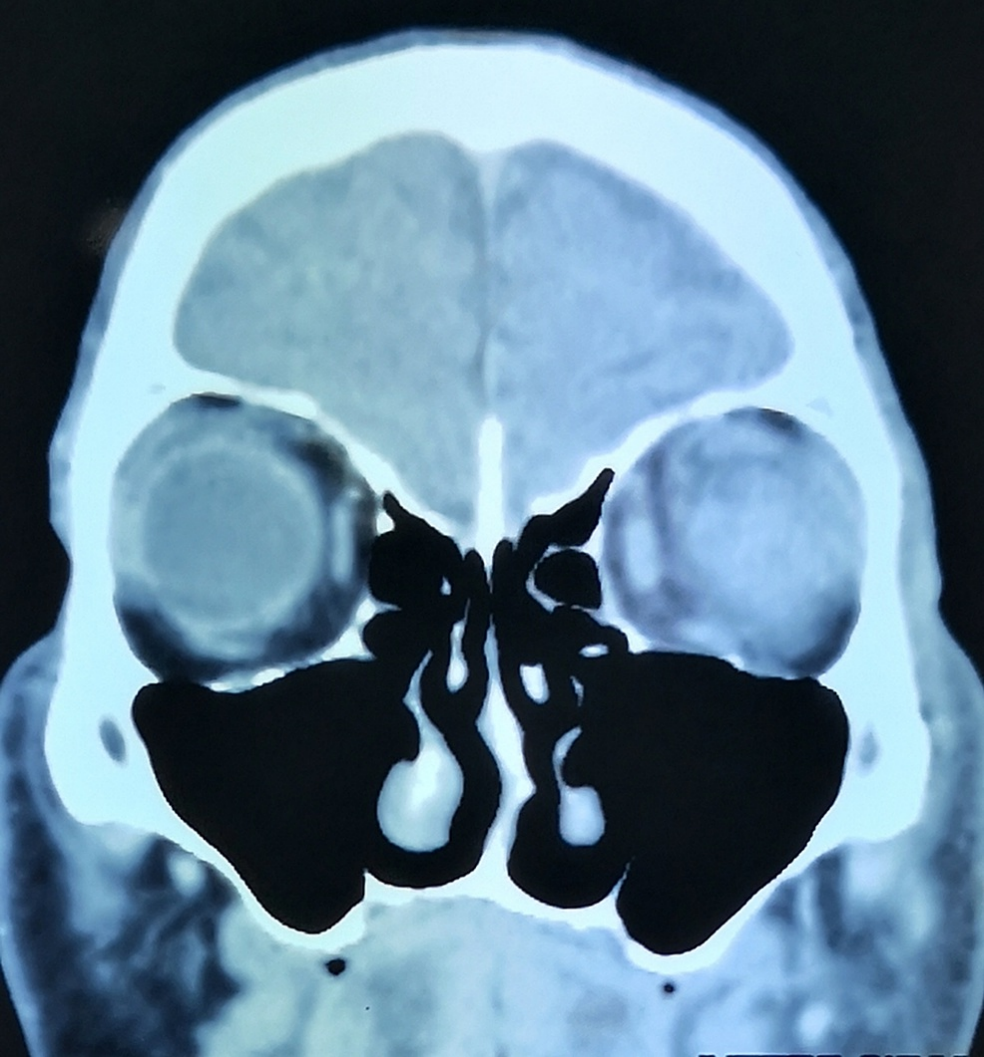
CT (coronal view) showing enhancement and thickening of the left eye scleral wall.

A 64-year-old gentleman who had no known comorbidity presented with sudden-onset, progressive left eye periorbital swelling. It was associated with blurring of vision, redness, and pain. His symptoms worsened rapidly, and he developed complete ptosis in the subsequent 24 hours. He had a low-grade fever three days prior to the development of ocular symptoms. His visual acuity was light perception, and a relative afferent pupillary defect was elicited in that eye. Ocular motility was restricted in all directions of gaze. Examination of the eye showed proptosis with severely chemosed conjunctiva, hazy cornea, and fibrin deposition in the anterior chamber (Figure 1). There was no view from funduscopic examination. B-scan later showed vitreous opacity with multiple loculations, as seen in Figure 2. An urgent CT scan revealed pre-septal and septal orbital wall thickening (Figure 3). The patient denied any history of trauma and was well premorbid. Common sources of endogenous panophthalmitis such as dental lesions and sinusitis were also ruled out.

**Figure 4:**
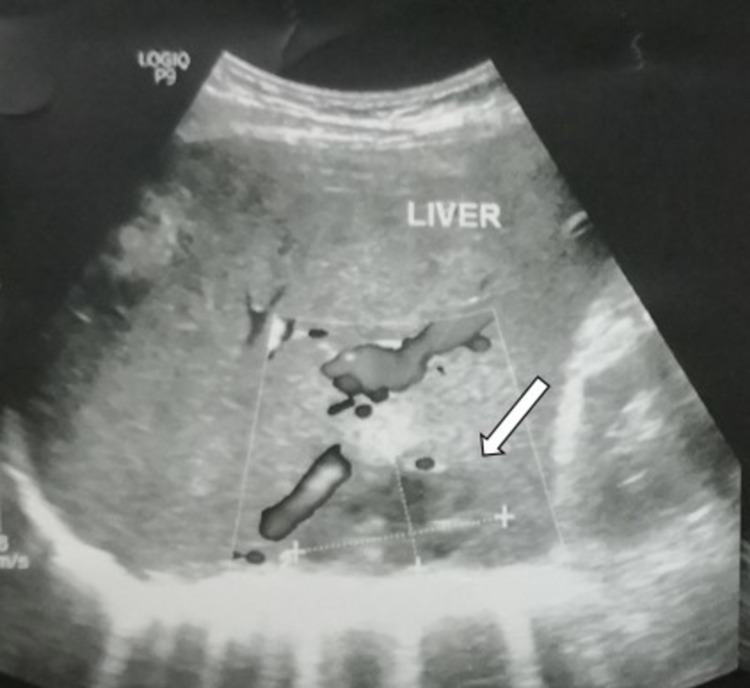
Ultrasound hepatobiliary system showing a hypoechoic lesion (arrow) in liver segment 6.

High-dose intravenous ceftriaxone and metronidazole were commenced, with concurrent intravitreal ceftazidime (2mg/0.1mL) and vancomycin (1mg/0.1mL) every 48 hours. All cultures from intravitreal taps were negative. Full blood count showed a raised white cell count of 18.6 × 10^9^/L, with neutrophils predominating. His total bilirubin level was 262umol/L, slightly raised above the normal range. Other parameters of his liver function and erythrocyte sedimentation rate were normal. An echocardiogram was normal, but ultrasound of the hepatobiliary system revealed a hypoechoic lesion in the right lobe of his liver, suggestive of a liver abscess, measuring 4.0 x 2.8 x 4.4 cm (Figure 4). Blood cultures grew *Klebsiella pneumoniae*, and hence a diagnosis of endogenous *Klebsiella pneumoniae* panophthalmitis was made. Surgical and infectious disease services were consulted to assist in the patient’s management.

**Figure 5:**
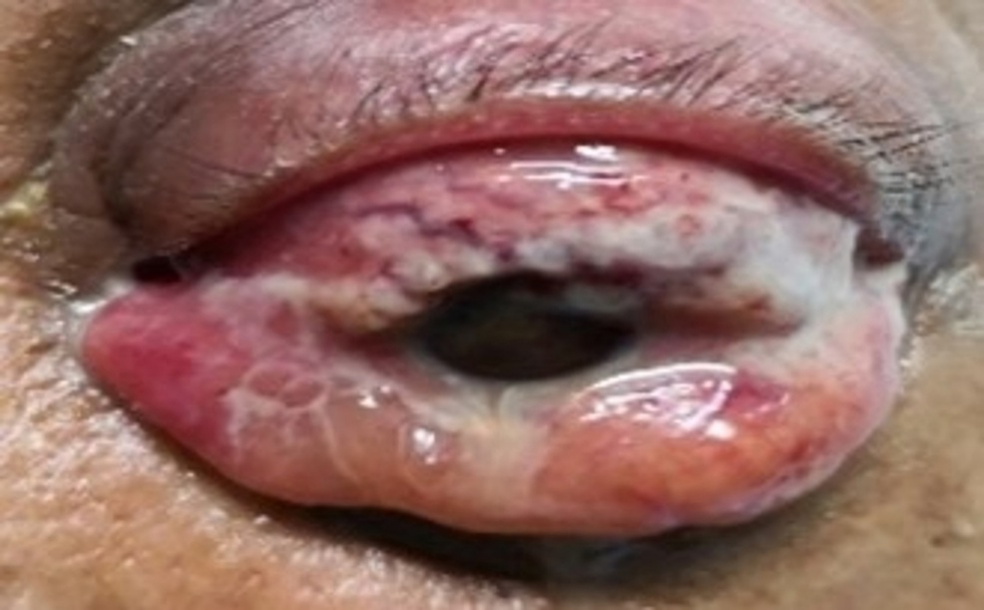
Day 8 of admission. Complete ophthalmoplegia with 360° conjunctival chemosis and pus discharge from the superior part of the conjunctiva can be seen.

**Figure 6:**
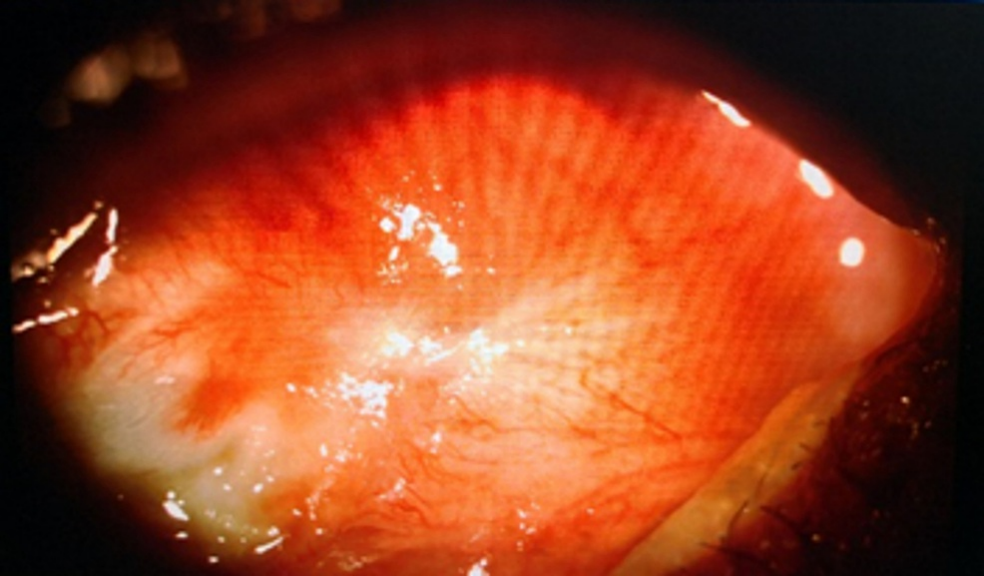
Three weeks post-evisceration. The conjunctiva had healed well spontaneously.

Unfortunately, his eye condition deteriorated despite the aggressive treatment. He developed complete ophthalmoplegia over the left globe. There was 360° of conjunctival chemosis, and pus was seen oozing from the superior conjunctiva (Figure 5). Evisceration of the eye was performed when we found scleral melting and globe perforation. In view of the active ongoing infection, the wound was not closed with the intention of secondary suturing later on. The patient completed high-dose intravenous ceftriaxone 2gm daily and metronidazole 500mg three times a day for two weeks, followed by oral Augmentin 625mg three times a day for four weeks. Ultrasound hepatobiliary system repeated two weeks after administration of intravenous antibiotics showed that the liver abscess had shrunk. At three weeks post-operatively, the wound had healed well without the need for secondary suturing (Figure 6).

## Discussion

Panophthalmitis is the most extensive manifestation of endophthalmitis and often results in poor visual outcome. It can be exogenous, when inoculated with pathogens following traumatic eye injury, post-operatively, or from eye infections, e.g., infective keratitis. On the other hand, organisms can metastasize through the hematogenous route to the eye from a distant focus of infection, resulting in endogenous panophthalmitis. Western and East Asian countries have shown distinct causative organisms for endogenous endophthalmitis [1]. Gram-positive organisms were reported as the commonest causative pathogens in Western countries, whereas gram-negative organisms are predominantly seen in cases from East Asian countries [2]. Studies have shown that liver abscesses are the most common infective source for endogenous endophthalmitis, with a prevalence of 0.8%-6.9% [3-5]. Other common sources include urinary tract infections, bacteremia, pneumonia, and soft tissue injuries [2].

*Klebsiella* pneumoniae is a gram-negative rod that commonly causes hepatobiliary, urinary tract, and nosocomial infections. It is the most common pathogen found in cases of endogenous endophthalmitis related to pyogenic liver abscesses in South East Asian countries [1]. Numerous reports regarding the high incidence of endogenous *Klebsiella* pneumoniae infection in countries such as Taiwan, South Korea, and Singapore have been published [4]. However, these studies commonly involved patients with comorbidities. This high incidence of *Klebsiella* infection is probably due to the genetic predisposition toward this infection in Asian populations, greater virulence of *Klebsiella* strain, and an overall increased antibiotic resistance to *Klebsiella pneumoniae* [1].

Patients with comorbidities such as diabetes mellitus, alcoholism, liver disease, systemic immunocompromised state, or previous usage of antibiotics are susceptible to *Klebsiella* pneumoniae infection [1,3,6]. Clinical signs of infection with this pathogen include rapidly progressive, severe intraocular inflammation, and extensive posterior segment involvement. Chen et al. had stated several risk factors for poor visual outcome in endogenous *Klebsiella* pneumoniae endophthalmitis, which include initial visual acuity worse than counting fingers, eye pain, hypopyon, ocular hypertension, positive intraocular fluid cultures, subretinal abscess, unilateral involvement, delayed ophthalmologist visit, initial presentation of ocular symptoms ahead of systemic symptoms, and corneal edema [5].

Panophthalmitis is more commonly seen in immunocompromised patients or patients with debilitating illness. Our case demonstrated the rapid and devastating sequelae of endogenous *Klebsiella* pneumoniae panophthalmitis in a gentleman with no comorbidities. Identifying the focus of infection in endogenous panophthalmitis can be challenging, such as in this case where the patient had a silent liver abscess. Early diagnosis and prompt treatment both systematically and locally to the eye are important to reduce morbidity and mortality in patients. The endogenous source of infection needs to be thoroughly investigated. Multimodal imaging including ultrasonography and computed tomography (CT) scans play an important role in the management, especially in our case where the patient presented only with eye symptoms. Failure to detect the primary source of endogenous panophthalmitis often causes a delay in treatment. A multidisciplinary approach is required in the case of endogenous panophthalmitis in order to provide the patient with comprehensive care. However, despite the efforts, the ocular outcome for this patient was poor.

Early systemic antibiotics in combination with intravitreal antibiotics remain the mainstay of treatment in endogenous panophthalmitis. Intravitreal injections are given as an adjunct to intravenous therapy in endogenous panophthalmitis in view of reduced permeability of the retinal pigmented epithelium to systemically administered drugs. Trans pars plana vitrectomy (TPPV) is usually performed in endophthalmitis cases especially in patients with uncontrolled infection and poor clinical response to intravitreal and systemic antibiotic administration. TPPV mechanically removes infective elements and inflammatory cells, as well as toxic debris from the vitreous cavity. It provides earlier visualization of the retina, allows intraocular fluid sampling for more precise microbiological analysis, and promotes greater penetration of antibiotics. Recently, many ophthalmologists have moved toward the trend of complete and early vitrectomy as an alternative to the Endophthalmitis Vitrectomy Study (EVS) recommendations, which is to perform TPPV only for patients presenting with visual acuity of light perception or less [7]. Several publications have demonstrated successful vision salvage after early TPPV [1,2,4,8]. However, there is no general consensus regarding TPPV in the management of endogenous panophthalmitis. Extensive inflammation, weak outer structure of the globe, and poor visualization of posterior segment of the eye are the challenges for vitrectomy in panophthalmitis cases. In many cases, evisceration or enucleation is required as a last sort of surgical treatment.

The role of steroids in endogenous panophthalmitis is still controversial. Previous studies suggested that there are some benefits in using intravitreal corticosteroids, as it produces favorable visual outcomes [1,5]. However, there is insufficient support in the current literature to recommend intravitreal corticosteroids as a standard of care. Finally, we would also like to emphasize the importance of proper wound dressing and care. Our patient was compliant with his wound dressing schedules. He achieved good wound healing without the need for secondary suturing.

## Conclusions

This case report demonstrates the devastating manifestation of *Klebsiella pneumoniae* infection in a healthy adult with no comorbidities. It also emphasizes the rapid and noxious outcomes of *Klebsiella pneumoniae* panophthalmitis, in our case developing from an asymptomatic liver abscess. Prompt diagnosis and aggressive management are warranted to prevent morbidity and mortality from this detrimental infection. A high index of this infection is necessary as it can be easily missed in healthy adults. Good wound dressing is a key management in post-evisceration wound care.
